# Simultaneous Analysis for Quality Control of Traditional Herbal Medicine, Gungha-Tang, Using Liquid Chromatography–Tandem Mass Spectrometry

**DOI:** 10.3390/molecules27041223

**Published:** 2022-02-11

**Authors:** Chang-Seob Seo, Hyeun-Kyoo Shin

**Affiliations:** KM Science Research Division, Korea Institute of Oriental Medicine, Daejeon 34054, Korea; hkshin@kiom.re.kr

**Keywords:** simultaneous analysis, quality control, Gungha-tang, LC–MS/MS

## Abstract

Gungha-tang (GHT), a traditional herbal medicine, consists of nine medicinal herbs (Cnidii Rhizoma, Pinelliae Tuber, Poria Sclerotium, Citri Unshius Pericarpium, Citri Unshius Pericarpium Immaturus, Aurantii Fructus Immaturus, Atracylodis Rhizoma Alba, Glycyrrhizae Radix et Rhizoma, and Zingiberis Rhizoma Recens). It has been used for various diseases caused by phlegm. This study aimed to develop and verify the simultaneous liquid chromatography–tandem mass spectrometry (LC–MS/MS) analysis method, using nine marker components (liquiritin apioside, neoeriocitrin, narirutin, naringin, hesperidin, neohesperidin, liquiritigenin, glycyrrhizin, and 6-shogaol) for quality control of GHT. LC–MS/MS analysis was conducted using a Waters TQ-XS system. All marker analytes were separated on a Waters Acquity UPLC BEH C_18_ column (2.1 × 100 mm, 1.7 μm) using gradient elution with a distilled water solution (containing 5 mM ammonium formate and 0.1% [*v*/*v*] formic acid)–acetonitrile mobile phase. LC–MS/MS multiple reaction monitoring (MRM) analysis was carried out in negative and positive ion modes of an electrospray ionization source. The developed LC–MS/MS MRM method was validated by examining the linearity, limits of detection and quantification, recovery, and precision. LOD and LOQ values of nine markers were calculated as 0.02–8.33 ng/mL and 0.05–25.00 ng/mL. The recovery was determined to be 89.00–118.08% and precision was assessed with a coefficient of variation value of 1.74–8.64%. In the established LC–MS/MS MRM method, all markers in GHT samples were detected at 0.003–16.157 mg/g. Information gathered during the development and verification of the LC–MS/MS method will be useful for the quality assessment of GHT and other herbal medicines.

## 1. Introduction

Traditional herbal medicines, traditional Korean medicines (TKMs), traditional Chinese medicines (TCMs), and Kampo medicines (KMs), characterized by multiple components and multiple targets, have long been used in Asian countries, especially Korea, China, and Japan, for the treatment of and protection against various diseases, and for maintaining health [1]. These TKMs, TCMs, and KMs consist of combinations of at least two or more medicinal herbs and are taken in the form of decoction [2,3].

Gungha-tang (GHT), one of these traditional herbal medicines, consists of nine medicinal herbs: Cnidii Rhizoma, Pinelliae Tuber, Poria Sclerotium, Citri Unshius Pericarpium, Citri Unshius Pericarpium Immaturus, Aurantii Fructus Immaturus, Atracylodis Rhizoma Alba, Glycyrrhizae Radix et Rhizoma, and Zingiberis Rhizoma Recens. GHT was first recorded in Ren Zhai Zhi Zhi Fang Lun written by Shiying Yang, a medical doctor from Southen Song Dynasty [4], and thereafter in Dong Eui Bo Gam written by Jun Heo during in the Joseon Dynasty. GHT is reported to be used for diseases caused by phlegm [5].

Nine herbal medicines have been reported to have various biological effects, for example, anti-inflammatory, antioxidant, anticancer, antitumor, antidiabetes, antiaging, anti-obesity, neuroprotective, and antibacterial activities [6,7,8,9,10,11,12,13,14]. Recently, a study on the safety of a single administration of GHT was reported by An et al. [4], but few studies on the biological activity have been reported.

The main ingredients of GHT, composed of nine herbs, are the following: chlorogenic acid, ferulic acid, senkyunolide A, and Z-ligustilide from Cnidii Rhizoma; homogentisic acid and 3,4-dihydroxybenzaldehyde from Pinelliae Tuber; pachymic acid, dehydrotumulosic acid, and polyporenic acid C from Poria Sclerotium; naringin, hesperidin, and narirutin from Citri Unshius Pericarpium, Citri Unshius Pericarpium Immaturus, and Aurantii Fructus Immaturus; atractylenolide I and III from Atracylodis Rhizoma Alba; liquiritin, liquiritin apioside, and glycyrrhizin from Glycyrrhizae Radix et Rhizoma; and 6-gingerol from Zingiberis Rhizoma Recens [15,16,17,18,19,20,21,22,23]. Analytical studies to determine qualitative, quantitative, or chemical profiling analyses have been conducted to evaluate the quality of each herb using high-performance liquid chromatography (HPLC) or liquid chromatography–tandem mass spectrometry (LC–MS/MS) systems for the main active components of each herbal medicine mentioned above [15,16,17,18,19,20,21,22,23]. However, no studies have been reported for the quality control of GHT composed of a combination of these nine herbal medicines.

To date, many researchers have used analytical techniques such as HPLC, LC–MS/MS, and gas chromatography–mass spectrometry for the quality control of complex formulations such as TKMs, TCMs, and KMs [24,25,26,27,28]. Among the various analytical techniques, the analytical methods that include HPLC and LC–MS/MS are currently the most widely used for standardization purposes. In particular, the sensitive, accurate, and reliable LC–MS/MS system is being used in standardization studies for numerous phytochemical components that constitute TKMs, TCMs, and KMs [29].

Therefore, in the present study, a simultaneous analysis method was developed, and then verified, utilizing the LC–MS/MS multiple reaction monitoring (MRM) assay, for efficient quality control of GHT using the following nine marker components: liquiritin apioside, neoeriocitrin, narirutin, naringin, hesperidin, neohesperidin, liquiritigenin, glycyrrhizin, and 6-shogaol.

## 2. Results and Discussion

### 2.1. Optimization of LC–MS/MS MRM Conditions

**Figure 1:**
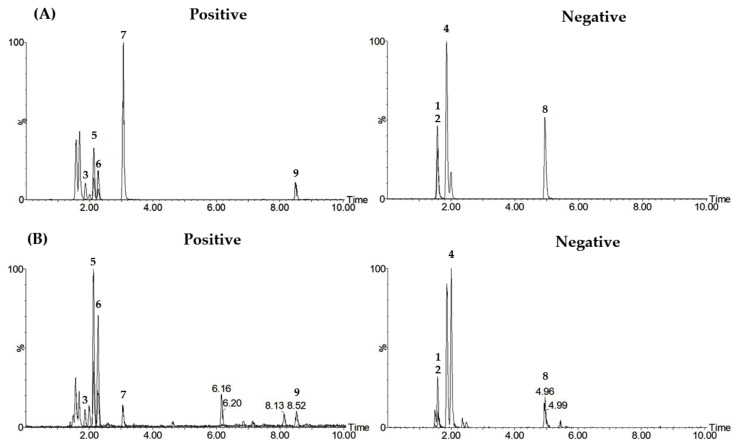
Total ion chromatograms of mixed standard solution of the nine marker components (**A**) and 70% methanolic solution of the freeze-dried GHT water decoction sample (**B**) acquired by LC–MS/MS MRM in positive and negative ion modes. Liquiritin apioside (1), neoeriocitrin (2), narirutin (3), naringin (4), hesperidin (5), neohesperidin (6), liquiritigenin (7), glycyrrhizin (8), and 6-shogaol (9).

For the quality assessment of GHT using the marker analytes, we first determined the optimal simultaneous determination conditions in LC–MS/MS MRM mode. Consequently, the nine markers (liquiritin apioside, neoeriocitrin, narirutin, naringin, hesperidin, neohesperidin, liquiritigenin, glycyrrhizin, and 6-shogaol) were separated using gradient elution with a distilled water solution (containing 5 mM ammonium formate and 0.1% [*v*/*v*] formic acid)–acetonitrile mobile phase system on an Acquity UPLC BEH C_18_ column (2.1 × 100 mm, 1.7 μm) maintained at 45 °C. Table 1 shows the optimal LC–MS/MS MRM parameters for the simultaneous quantification of each marker component. The established assay was successfully applied to the GHT sample and all markers were detected within 10 min, as shown in Figure 1 and Table 1.

### 2.2. Identification of Each Marker Analyte for LC–MS/MS MRM Analysis

In the LC–MS/MS MRM analysis of each marker analyte, use was made of an electrospray ionization source of negative and positive ion modes. Four components (liquiritin apioside, neoeriocitrin, naringin, and glycyrrhizin) were detected at *m*/*z* 549.3, 595.5, 579.3, and 821.9, respectively, in the form of [M–H]^−^, negative ion mode, and the remaining five components (naritutin, hesperidin, neohesperidin, liquiritigenin, and 6-shogaol) were detected at *m*/*z* 581.0, 611.5, 611.0, 257.2, and 277.2, respectively, in the form of [M+H]^+^, positive ion mode (Figure 1, Table 1). As MRM conditions for LC–MS/MS simultaneous analysis, the precursor ion (Q1) and product ion (Q3) peaks for each marker analyte were set as shown in Table 1. Liquiritin apioside, a flavanone, detected a Q3 ion peak at *m*/*z* 255.0 (M–H–Glc-Api]^−^), generated by removal of the glucosyl-apisyl group [30]. Q3 ion peaks of naritutin, naringin, hesperidin, and neohesperidin were detected at *m*/*z* 273.0 ([M+H–Glc-Rham]^+^), 271.0 ([M–H–Glc-Rham]^−^), 303.2 ([M+H–Glc-Rham]^+^), and 303.0 ([M+H–Glc-Rham]^+^), respectively, in the form of an aglycone from which rutinose was eliminated [31,32,33]. Neoeriocitrin was detected at *m*/*z* 151.0 in the form of [^1,3^A_0_–H]^−^, generated by retro-Diels–Alder (RDA) fragmentation of aglycone, from which rutinose had been removed [31,32]. The fragmentation of liquiritigenin is similar to that of neoeriocitrin; the Q3 ion peak was detected at *m*/*z* 137.0 ([M+H–4-vinylphenol]^+^) by RDA cleavage [31]. Glycyrrhizin was in the form of [di-GlcA–H]^−^, in which aglycone was lost, and a Q3 ion peak was detected at *m*/*z* 351.2 [30]. In 6-shogaol, the Q3 ion peak was detected at *m*/*z* 137.1 in the form of [M+H–C_9_H_15_O]^+^, by cleavage of the C1–C2 bond by the ketone functional group of the alkyl chain [34,35]. MS fragmentation for the simultaneous determination of each marker as described above is shown in Figure S2.

### 2.3. Method Validation of the Developed Analytical Method

In this research, the newly developed LC–MS/MS MRM analytical method for the simultaneous determination of the nine marker analytes in GHT samples was validated by evaluating the linearity, limit of detection (LOD), limit of quantification (LOQ), recovery, and precision. Table 2, Table 3, Table 4 show the results for various validation parameters. Briefly, the coefficient of determination (*r*^2^) value, which means the linearity of the calibration curve prepared in different concentration ranges for each marker analyte, was 0.9950–0.9968, showing good linearity, and LOD and LOQ values were estimated to be 0.02–8.33 ng/mL and 0.05–25.00 ng/mL, respectively (Table 2). The recovery test, calculated from Equation (1), was conducted to evaluate the accuracy of the developed method; it was determined to be 89.00–118.08% (Table 3). The acceptance criteria for recovery test for validation of analysis of traditional herbal medicines such as TKMs, TCMs, and KMs are generally accepted with ±20%, so results of our study show that they are suitable [36,37]. In the precision verification, the repeatability of the retention time and peak area of the marker analytes was evaluated as the coefficient of variation (CV) values (calculated from Equation (2)); it was determined to be 0.08–0.52% and 3.04–9.64%, respectively (Table S2). In addition, the CV (%) values of intra- and interday precisions of the nine marker analytes were also determined to be <10.00% (Table 4). From the above various verification results, indications are that the LC–MS/MS MRM assay developed in this study is suitable and appropriate as an analytical method for the quality evaluation of GHT.

### 2.4. Simultaneous Determination of the Nine Marker Analytes in GHT Samples Using the Developed LC–MS/MS MRM Assay

Simultaneous determination of the nine marker analytes in GHT samples was conducted using the LC–MS/MS MRM assay developed and validated in this study. All marker analytes (liquiritin apioside, neoeriocitrin, narirutin, naringin, hesperidin, neohesperidin, liquiritigenin, glycyrrhizin, and 6-shogaol) were eluted, at 1.57, 1.58, 1.86, 1.99, 2.13, 2.27, 3.05, 4.95, and 8.50 min, respectively (Table 1 and Figure S3). The nine marker analytes in the lyophilized GHT samples were detected at 0.003–16.157 mg/g. The detailed content of each marker compound is given in Table 5. Among these markers, narirutin, narigin, hesperidin, and neohesperidin, derived from Citri Unshius Pericarpium, Citri Unshius Pericarpium Immaturus, Aurantii Fructus Immaturus, were present in large amounts. These results suggest the possibility of them being useful as basic data for the analysis of quality assessment of GHT.

## 3. Materials and Methods

### 3.1. Plant Materials

Nine raw herbal medicines constituting GHT were purchased from Kwangmyungdang Medicinal Herbs (KMH; Ulsan, Korea), a herbal medicine supplier for pharmaceuticals, in November 2017. All medicinal herbs were used after morphological verification by Dr. Seung-Yeol Oh, president of KMH. Detailed information on all raw herbs is shown in Table S1. Specimens of the nine raw herbal medicines (2017KE58–1 to 2017KE58–5) were deposited at the KM Science Research Division, Korea Institute of Oriental Medicine.

### 3.2. Chemicals and Reagents

The nine standard compounds used as markers for quality assessment of GHT in this study are shown in Figure S1. These compounds were provided by commercial suppliers and used for LC–MS/MS analysis: liquiritin apioside (C_26_H_30_O_13_, CAT No. DR10690, 99.6%), hesperidin (C_28_H_34_O_15_, CAT No. DR10882, 98.7%), neohesperidin (C_28_H_34_O_15_, CAT No. DR10883, 98.4%), and 6-shogaol (C_17_H_24_O_3_, CAT No. DR10924, 99.2%) from Shanghai Sunny Biotech Co., Ltd. (Shanghai, China); neoeriocitrin (C_27_H_32_O_15_, CAT No. TBW00746, 99.9%) from Wuhan ChemNorm Biotech Co., Ltd. (Wuhan, China); narirutin (C_27_H_32_O_14_, CAT No. BP0985, 99.5%), liquiritigenin (C_15_H_12_O_4_, CAT No. BP0873, 99.8%), and glycyrrhizin (C_42_H_62_O_16_, CAT No. BP0682, 99.1%) from Chengdu Biopurify Phytochemicals Ltd. (Chengdu, China); naringin (C_27_H_32_O_14_, CAT No. 71162, 95.0%) from KGaA (Darmstadt, Germany). Methanol and acetonitrile were LC–MS grade and supplied by ThermoFisher Scientific (San Jose, CA, USA). Purified water was used, specifically, produced through a Vivagen water purification system (EXL3 Analysis 16, Seongnam, Korea). Formic acid (≥99.5%) was supplied by Fujifilm Wako Pure Chemical Co., Ltd. (Osaka, Japan) and ammonium formate (99.0%) by Kanto Chemical Co., Inc. (Tokyo, Japan).

### 3.3. Preparation of GHT Water Sample

A sample of GHT in water was prepared following the same protocol as that in other previously reported methods for preparing a herbal prescription [38] (see Table S1). After mixing nine herbal medicines (Cnidii Rhizoma, Pinelliae Tuber, and Poria Sclerotium, each 881.52 g; Citri Unshius Pericarpium, Citri Unshius Pericarpium Immaturus, and Aurantii Fructus Immaturus, each 441.94 g; Atracylodis Rhizoma Alba and Glycyrrhizae Radix et Rhizoma, each 220.97 g; and Zingiberis Rhizoma Recens, 587.68 g), in a weight ratio (*w*/*w*), 50 L of distilled water was added, and extraction was performed under pressure (0.98 bar) at 100 °C for 2 h. The extract was subsequently filtered through a sieve (53 μm mesh) and then freeze dried (PVTFD100R, IlShinBioBase, Yangju, Korea) to afford a powder sample of about 1.0 kg (yield 20.1%).

### 3.4. Preparation of Samples and Standard Solutions for LC–MS/MS Quantification of the Nine Marker Analytes in GHT Samples

To analyze simultaneously the nine marker analytes in a GHT sample using LC–MS/MS, 70% methanol was added to approximately 50 mg of the lyophilized GHT sample to make up a volume of 50 mL. The mixed sample solution was continuously subjected to ultrasonic extraction for 5 min and vortexing for 1 min. Prior to analysis, the prepared sample solution was diluted 50-fold with 70% methanol and filtered through a hydrophobic polytetrafluoroethylene membrane filter (0.2 μm; Pall Life Sciences, Ann Arbor, MI, USA).

A standard solution for each marker analyte was prepared at a concentration of 100.0 μg/mL, using methanol, and then stored at 4 °C until analysis.

### 3.5. LC–MS/MS Instrumentation and Experimental Conditions for Simultaneous Determination of the Nine Marker Analytes in GHT Samples

Simultaneous determination of the nine marker analytes in GHT samples by LC–MS/MS was conducted using a previously reported protocol [39,40]. Briefly, LC–MS/MS analysis was performed using a Waters Acquity UPLC system (Milford, MA, USA) coupled with a Waters Xevo TQ-XS triple quadrupole MS system. Markers were separated on an Waters Acquity UPLC BEH C_18_ column (2.1 × 100 mm, 1.7 μm), maintained at 45 °C, using gradient elution with a distilled water solution (containing 5 mM ammonium formate and 0.1% [*v*/*v*] formic acid)–acetonitrile mobile phase system. Detailed experimental parameters of ultra-performance liquid chromatography and MS for simultaneous determination are summarized in Table S3.

### 3.6. Method Validation of the Developed LC–MS/MS MRM Assay

The simultaneous LC–MS/MS analysis method developed for the consistent quality control of GHT was verified by evaluating linearity, LOD, LOQ, accuracy (recovery), and precision (repeatability, intraday precision, and interday precision). Verification of linearity was evaluated by the *r*^2^ of the calibration curve prepared at different concentrations of each marker analyte: 25.00–400.00 ng/mL (liquiritin apioside), 50.00–800.00 ng/mL (neoeriocitrin, narirutin, naringin, hesperidin, and glycyrrhizin), 100.00–1600.00 ng/mL (neohesperidin), and 0.10–1.60 ng/mL (liquiritigenin and 6-shogaol). LOD and LOQ were automatically calculated by the LC–MS/MS system (MassLynx software, version 4.2, Waters, Milford, MA, USA) as a signal-to-noise ratios of 3 and 10, respectively.

The accuracy verification of the newly developed LC–MS/MS method was performed through the recovery test. In other words, the recovery (%) was determined by adding known concentrations of each standard marker analyte (low, medium, and high) to the GHT sample as shown in Table 3, and calculated from Equation (1).

(1) $$\mathrm{Recovery}\;\left(\%\right)=\frac{\mathrm{amount}\;\mathrm{found}}{\mathrm{spiked}\;\mathrm{amount}}\times 100$$

The precision (repeatability, intraday precision, and interday precision) of our newly developed analytical method was evaluated by calculating the CV value of each parameter. The repeatability was validated by calculating the CV value of retention time and peak area of each marker analyte, after six measurements, using a standard solution. Intraday precision and interday precision were assessed with CV values calculated after measurements for within day and 3 consecutive days on the three concentrations, respectively. The CV (%) value was calculated from Equation (2).

(2) $$\mathrm{CV}\;\left(\%\right)=\frac{\mathrm{standard}\;\mathrm{deviation}\;\left(\mathrm{SD}\right)}{\mathrm{mean}}\times 100$$

### 3.7. Statistical Analysis

Data were expressed as mean, SD, and CV (%) using Microsoft Excel 2019 software (Microsoft Co., Redmond, WA, USA).

## 4. Conclusions

In this study, for the first time, a sensitive, accurate, and reliable LC–MS/MS MRM assay for efficient quality assessment of GHT, a traditional herbal prescription, was developed using nine selected marker analytes. The developed assay was evaluated by examining the linearity, LOD, LOQ, accuracy, and precision. The established LC–MS/MS MRM assay is expected to be useful in not only the efficient quality control of GHT, but also in further studies on other TKMs, TCMs, and KMs.

## Figures and Tables

**Table 1:** LC–MS/MS MRM conditions for simultaneous determination of marker compounds in GHT.

Compound	Ion Mode	Molecular Weight	Precursor Ion (Q1)	Product Ion (Q3)	Cone Voltage (V)	Collision Energy (eV)	Retention Time (min)
Liqiritin apioside	−	550.2	549.3	255.0	45	30	1.57
Neoeriocitrin	−	596.2	595.5	151.0	30	40	1.58
Narirutin	+	580.2	581.0	273.0	15	15	1.86
Naringin	−	580.2	579.3.	271.0	45	30	1.99
Hesperidin	+	610.2	611.5	303.2	20	15	2.13
Neohesperidin	+	610.2	611.0	303.0	15	20	2.27
Liquiritigenin	+	256.1	257.2	137.0	35	35	3.05
Glycyrrhizin	−	822.4	821.9	351.2	45	40	4.95
6-Shogaol	+	276.2	277.2	137.1	25	15	8.50

**Table 2:** Various parameters for simultaneous determination of marker analytes in GHT using the LC–MS/MS MRM assay.

Analyte	Linear Range (ng/mL)	Regression Equation ^a^ y=ax+b	*r* ^2^	LOD (ng/mL)	LOQ (ng/mL)
Liqiritin apioside	25.00–400.00	*y* = 68.42*x* + 52.85	0.9968	8.33	25.00
Neoeriocitrin	50.00–800.00	*y* = 48.69*x* − 80.24	0.9958	0.83	2.50
Narirutin	50.00–800.00	*y* = 20.32*x* + 202.74	0.9954	3.33	10.00
Naringin	50.00–800.00	*y* = 23.58*x* − 107.24	0.9950	8.33	25.00
Hesperidin	50.00–800.00	*y* = 153.88*x* + 509.37	0.9951	1.67	5.00
Neohesperidin	100.00–1600.00	*y* = 29.03*x* + 905.96	0.9950	0.33	1.00
Liquiritigenin	0.10–1.60	*y* = 19,647.00*x* + 239.15	0.9959	0.02	0.05
Glycyrrhizin	50.00–800.00	*y* = 14.51*x* − 40.59	0.9953	1.67	5.00
6-Shogaol	0.10–1.60	*y* = 19,566.10*x* + 659.29	0.9966	0.02	0.05

^a^*y*: peak area of each analyte; *x*: concentration of each analyte.

**Table 3:** Recovery tests for each marker analyte in GHT using the developed LC–MS/MS MRM assay.

Analyte	Spiked Amount (ng/mL)	Amount Found (ng/mL)	Recovery (%)	SD	CV (%)
Liquiritin apioside	200.00	234.34	117.17	1.44	1.23
	400.00	458.24	114.56	3.30	2.88
	800.00	862.88	107.86	0.78	0.72
Neoeriocitrin	50.00	59.04	118.08	1.31	1.11
	100.00	110.50	110.50	4.10	3.71
	200.00	219.08	109.54	3.74	3.41
Narirutin	500.00	557.52	111.50	2.36	3.02
	1000.00	1050.68	105.07	3.17	1.30
	2000.00	2010.86	100.54	1.31	1.11
Naringin	500.00	573.90	114.78	2.84	2.47
	1000.00	1157.74	115.77	2.10	1.81
	2000.00	2237.84	111.89	1.97	1.76
Hesperidin	500.00	544.38	108.88	1.75	1.61
	1000.00	1013.86	101.39	2.72	2.68
	2000.00	1983.78	99.19	1.72	1.73
Neohesperidin	1000.00	1076.62	107.66	7.19	6.68
	2000.00	2009.60	100.48	5.05	5.03
	4000.00	4121.26	103.03	3.02	2.94
Liquiritigenin	4.00	3.66	91.50	7.42	8.11
	8.00	7.88	98.50	6.75	6.86
	16.00	14.50	90.63	7.51	8.29
Glycyrrhizin	500.00	579.68	115.94	3.42	2.95
	1000.00	1173.10	117.31	2.13	1.81
	2000.00	2174.32	108.72	1.38	1.26
6-Shogaol	1.00	0.98	98.00	8.37	8.54
	2.00	1.90	95.00	7.91	8.32
	4.00	3.56	89.00	7.62	8.57

**Table 4:** Precision data for simultaneous determination of the nine marker analytes in the developed LC–MS/MS MRM assay.

Analyte	Conc. (ng/mL)	Intraday (*n* = 5)			Interday (*n* = 5)		
		Obtained Conc. (ng/mL)	Precision (%) ^a^	Accuracy (%)	Obtained Conc. (ng/mL)	Precision (%)	Accuracy (%)
Liquiritin apioside	200.00	180.82	3.58	90.41	201.70	2.32	100.85
	400.00	385.94	3.64	96.49	407.92	3.21	101.98
	800.00	784.44	4.66	98.06	807.92	2.45	100.99
Neoeriocitrin	50.00	44.56	5.35	89.12	50.08	3.34	100.15
	100.00	96.76	5.10	96.76	99.12	4.96	99.12
	200.00	204.04	2.10	102.02	203.84	2.62	101.92
Narirutin	500.00	464.20	6.88	92.84	502.50	3.86	100.50
	1000.00	940.26	4.62	94.03	976.10	3.51	97.61
	2000.00	1673.18	8.45	83.66	1868.60	3.92	93.43
Naringin	500.00	470.92	2.57	94.18	500.80	2.23	100.16
	1000.00	972.82	2.11	97.28	1008.50	1.89	100.85
	2000.00	2020.58	1.74	101.03	2019.00	1.85	100.95
Hesperidin	500.00	465.32	2.39	93.06	492.55	2.43	98.51
	1000.00	939.70	3.16	93.97	967.20	2.74	96.72
	2000.00	1982.78	3.06	99.14	1967.20	1.85	98.36
Neohesperidin	1000.00	955.86	5.67	95.59	987.40	5.54	98.74
	2000.00	1948.32	5.38	97.42	1929.40	5.04	96.47
	4000.00	3908.70	3.86	97.72	3997.20	3.05	99.93
Liquiritigenin	4.00	3.74	4.06	93.50	3.55	5.76	88.83
	8.00	7.30	3.21	91.25	7.77	4.85	97.08
	16.00	14.98	5.41	93.63	15.27	5.87	95.46
Glycyrrhizin	500.00	511.30	5.58	102.26	517.31	3.36	103.46
	1000.00	971.88	3.86	97.19	1027.81	2.62	102.78
	2000.00	1976.92	3.40	98.85	2031.49	2.34	101.57
6-Shogaol	1.00	0.98	8.54	98.00	0.92	8.64	92.00
	2.00	1.74	6.55	87.00	1.87	7.72	93.67
	4.00	3.88	8.43	97.00	4.05	6.17	101.17

^a^ Precision (%) is expressed as CV (%) calculated from Equation (2).

**Table 5:** Amounts of the nine marker analytes in GHT samples by LC–MS/MS MRM assay (*n* = 3).

Analyte	Amount					
	GHT-1 ^a^			GHT-2 ^b^		
	Mean (mg/g)	SD (×10^−1^)	CV (%)	Mean (mg/g)	SD (×10^−1^)	CV (%)
Liqiritin apioside	0.007	0.002	2.831	0.003	0.002	8.377
Neoeriocitrin	1.089	0.259	2.377	0.315	0.292	9.274
Narirutin	5.878	2.395	4.075	0.944	0.838	8.870
Naringin	16.157	1.297	0.803	2.785	2.473	8.880
Hesperidin	8.002	1.647	2.058	6.559	1.031	1.573
Neohesperidin	8.338	5.982	7.175	0.044	0.035	7.857
Liquiritigenin	2.423	0.518	2.139	0.809	0.741	9.160
Glycyrrhizin	6.416	0.818	1.275	2.801	0.819	2.923
6-Shogaol	0.007	0.007	9.042	0.008	0.007	8.072

^a^ GHT-1: sample was prepared in Korea Institute of Oriental Medicine; ^b^ GHT-2: sample was made by commercial pharmaceutical company.

## Data Availability

All data can be found in this paper.

## Supplementary Materials

The following supporting information can be downloaded, Figure S1: Chemical structures of the nine marker components in GHT; Figure S2: MS fragmentation of each marker analyte; Figure S3: Extracted ion chromatograms of each reference standard (A) and GHT sample (B) measured by LC–MS/MS MRM mode; Table S1: Composition of prepared GHT; Table S2: Repeatability of the nine marker analytes in the LC–MS/MS MRM assay (*n* = 6); Table S3: LC–MS/MS MRM experimental conditions for simultaneous determination of the nine marker analytes in GHT samples.
