# Supplementary material for: Simultaneous Analysis for Quality Control of Traditional Herbal Medicine, Gungha-Tang, Using Liquid Chromatography–Tandem Mass Spectrometry
Source: Molecules. 2022 Feb 11;27(4):1223. doi: 10.3390/molecules27041223 (PMC8877009; doi:10.3390/molecules27041223)
Supplement: Supplementary file 1 [file molecules-27-01223-s001.zip › molecules-1576664-supplementary.pdf]

molecules

MDPI

Article

Simultaneous Analysis for Quality Control of Traditional Herbal Medicine, Gungha-Tang, Using Liquid Chromatography–Tandem Mass Spectrometry

Table S1. Composition of prepared GHT.

Herbal medicine	Scientific name	Family	Using part	Origin	Amount (g)	Ratio (%)
Cnidii Rhizoma	*Cnidium officinale* Mak.	Umbelliferae	Rhizome	Yeongyang, Korea	881.52	17.63
Pinelliae Tuber	*Pinellia ternate* (Thunb.) Mak.	Araceae	Tuber	China	881.52	17.63
Poria Sclerotium	*Poria cocos* Wolf	Polyporaceae	Sclerotium	Bonghwa, Korea	881.52	17.63
Citri Unshius Pericarpium	*Citrus unshiu* Marcow.	Rutaceae	Peel	Jeju, Korea	441.94	8.84
Citri Unshius Pericarpium Immaturus	*Citrus unshiu* Marcow.	Rutaceae	Peel	China	441.94	8.84
Aurantii Fructus Immaturus	*Citrus aurantium* L.	Rutaceae	Fruit	China	441.94	8.84
Atracylodis Rhizoma Alba	*Atractylodes japonica* Koidz. Ex Kitam.	Compositae	Rhizome	Uljin, Korea	220.97	4.42
Glycyrrhizae Radix et Rhizoma	*Glycyrrhiza uralensis* Fisch.	Leguminosae	Root and rhizome	China	220.97	4.42
Zingiberis Rhizoma Recens	*Zingiber officinale* Rosc.	Zingiberaceae	Rhizome	Seosan, Korea	587.68	11.75
				Total	5000.0	100.0

Table S2. Repeatability of the nine marker analytes in the developed LC–MS/MS MRM assay (n = 6).

Analyte	CV (%) of retention time	CV (%) of peak area
Liqiritin apioside	0.52	3.11
Neoeriocitrin	0.26	4.36
Narirutin	0.40	8.17
Naringin	0.26	5.21
Hesperidin	0.19	4.07
Neohesperidin	0.28	9.64
Liquiritigenin	0.13	4.77
Glycyrrhizin	0.08	3.04
6-Shogaol	0.14	9.26

Table S3. LC–MS/MS MRM experimental conditions for simultaneous determination of the nine marker analytes in GHT samples.

UPLC conditions				MS conditions	
UPLC system	Acquity UPLC H-Class			MS system	Xevo TQ-XS
Column	Acquity UPLC BEH C_{18} column (2.1 mm × 100 mm, 1.7 μm)			MS software	MassLynx (version 4.2)
Column temp.	45 °C			Ionization mode	ESI^+ / ESI^-
Sample temp.	5 °C			Acquisition mode	MRM
Injection volume	2.0 μL			Capillary voltage	3.0 kV
Flow rate	0.3 mL/min			Cone gas flow	50 L/h
Mobile phase A	Distilled water (5 mM ammonium formate and 0.1% [v/v] formic acid)			Desolvation gas flow	700 L/h
Mobile phase B	Acetonitrile			Desolvation temp.	500 °C
Gradient elution	Time (min)	A (%)	B (%)	Source temp.	150 °C
	0.00	80	20		
	14.00	5	95		
	14.29	0	100		
	15.71	80	20		
	20.00	80	5		

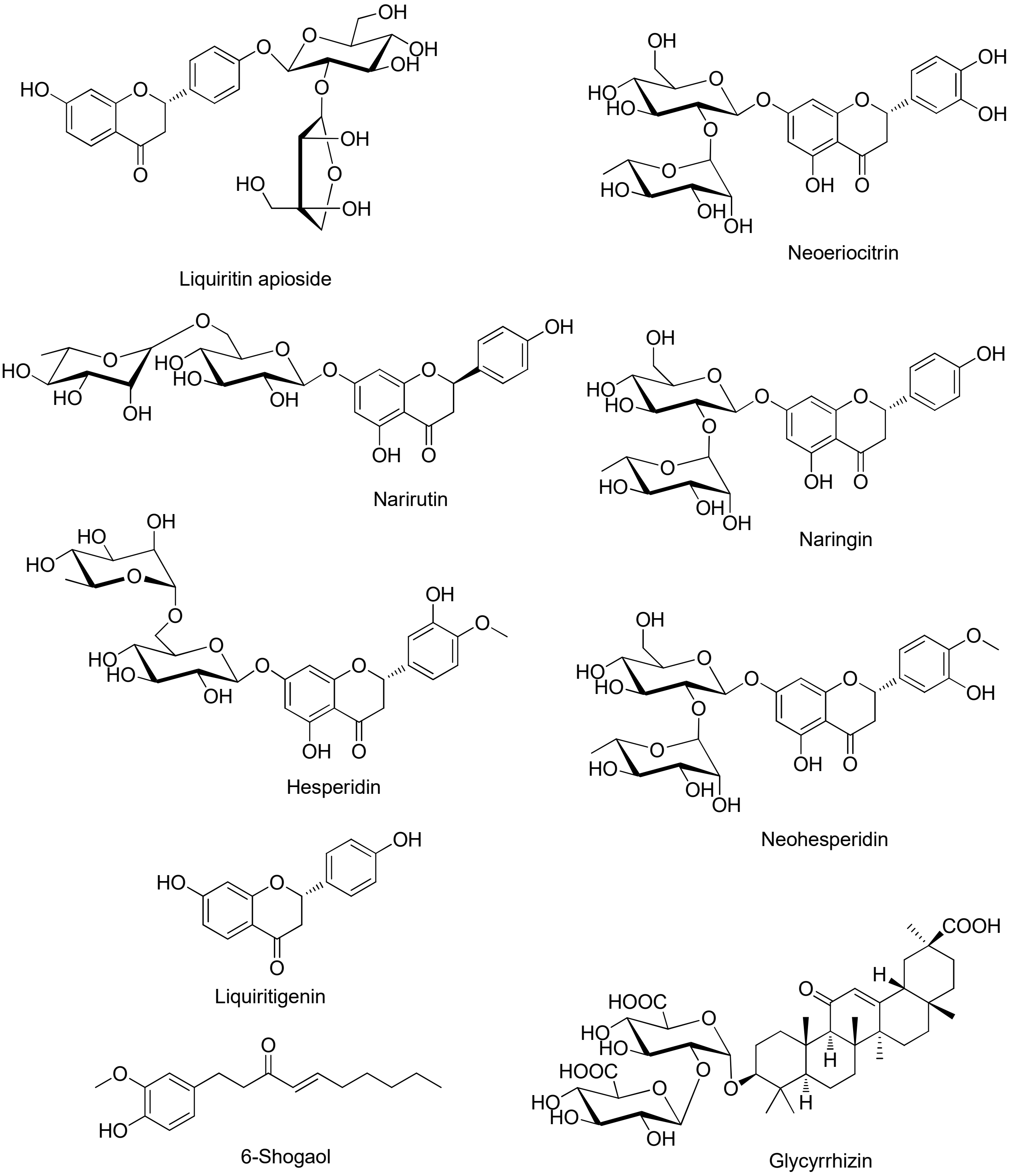

Figure S1. Chemical structures of the nine marker components in GHT.

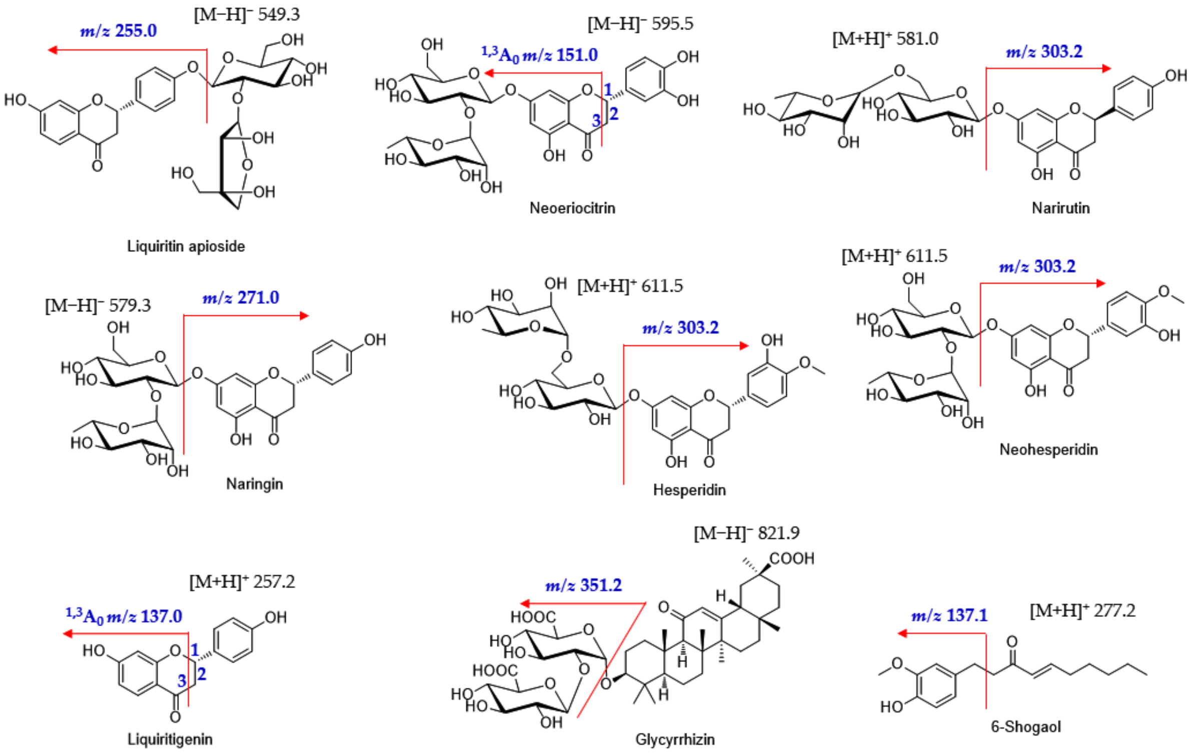

Figure S2. MS fragmentation of each marker analyte

(A)

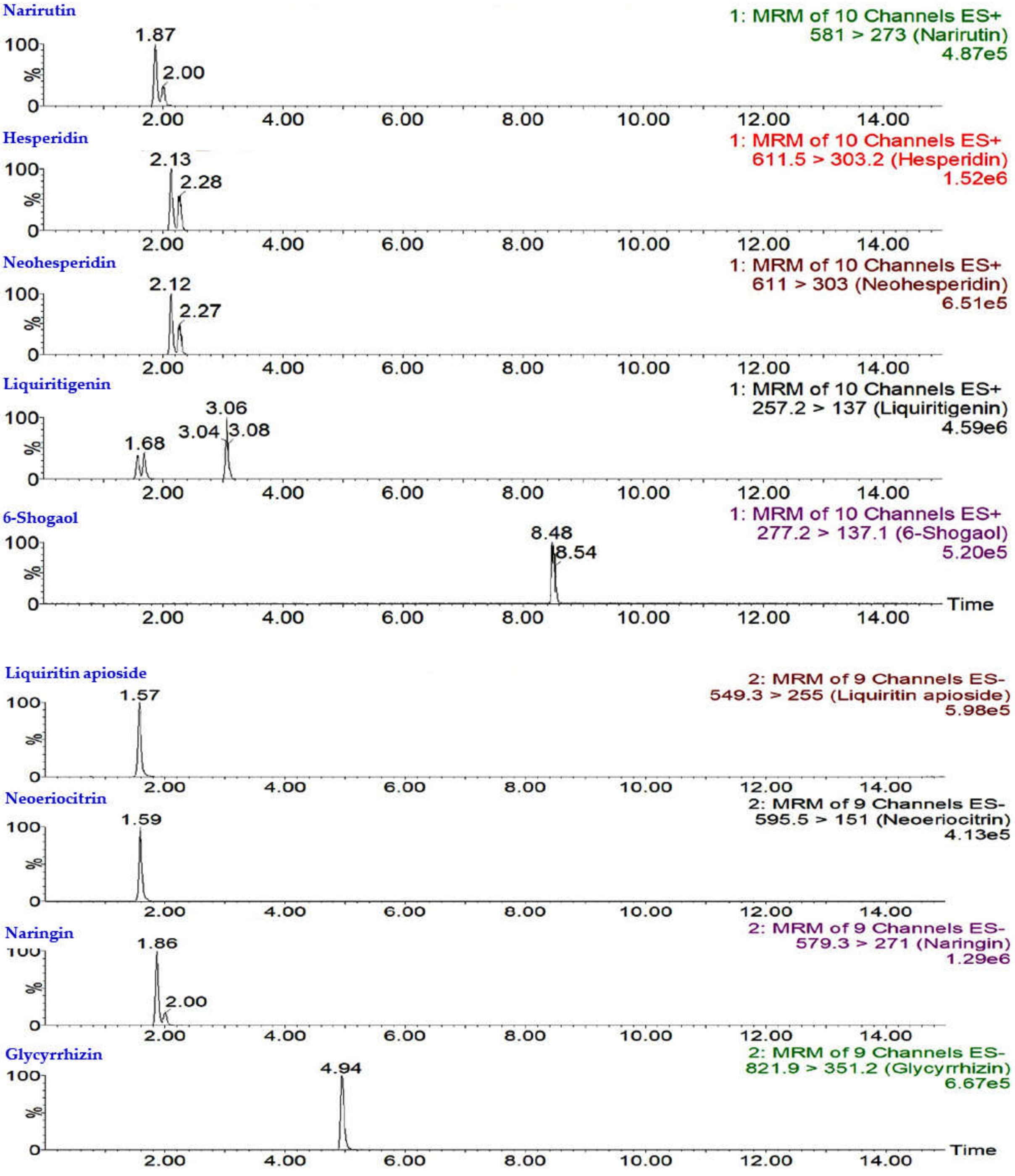

Narirutin
1: MRM of 10 Channels ES+
581 > 273 (Narirutin)
4.87e5
1.87
2.00
Hesperidin
1: MRM of 10 Channels ES+
611.5 > 303.2 (Hesperidin)
1.52e6
2.13
2.28
Neohesperidin
1: MRM of 10 Channels ES+
611 > 303 (Neohesperidin)
6.51e5
2.12
2.27
Liquiritigenin
1: MRM of 10 Channels ES+
257.2 > 137 (Liquiritigenin)
4.59e6
1.68
3.04
3.06
3.08
6-Shogaol
1: MRM of 10 Channels ES+
277.2 > 137.1 (6-Shogaol)
5.20e5
8.48
8.54
Liquiritin apioside
2: MRM of 9 Channels ES-
549.3 > 255 (Liquiritin apioside)
5.98e5
1.57
Neoeriocitrin
2: MRM of 9 Channels ES-
595.5 > 151 (Neoeriocitrin)
4.13e5
1.59
Naringin
2: MRM of 9 Channels ES-
579.3 > 271 (Naringin)
1.29e6
1.86
2.00
Glycyrrhizin
2: MRM of 9 Channels ES-
821.9 > 351.2 (Glycyrrhizin)
6.67e5
4.94
100
%
0
2.00
4.00
6.00
8.00
10.00
12.00
14.00
Time

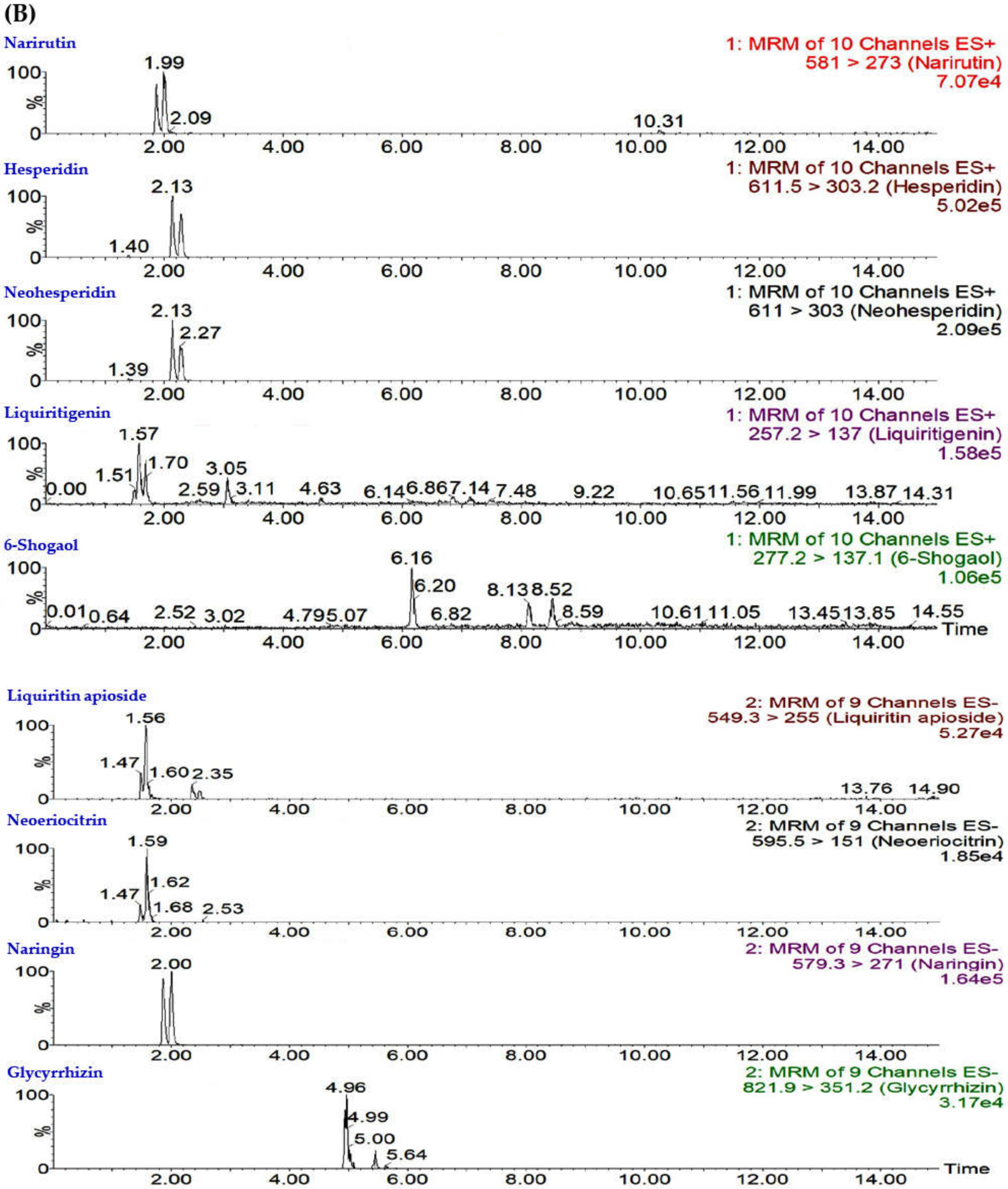

Figure S3. Extracted ion chromatograms of each reference standard (A) and GHT sample (B) measured by LC–MS/MS MRM mode.
